# Perforation of pancreatic pseudocyst diagnosed with endoscopy and treated with percutaneous drainage

**DOI:** 10.1002/deo2.295

**Published:** 2023-09-12

**Authors:** Mako Koseki, Yusuke Hashimoto

**Affiliations:** ^1^ Department of Medicine Icahn School of Medicine at Mount Sinai Mount Sinai Beth Israel New York USA; ^2^ Department of Internal Medicine Division of Gastroenterology University of Florida Jacksonville USA

**Keywords:** fluid collection, LAMS, pancreatitis, perforation, pseudocyst

## Abstract

Perforation is a rare but fatal complication of pancreatic pseudocysts. It is generally diagnosed by computed tomography imaging with hemorrhagic ascites and pneumoperitoneum. Traditionally, surgery was the mainstream for treating this critical state. Recently, alternative therapies have also been deemed useful. Herein, we describe the case of a 54‐year‐old with perforation of pancreatic pseudocyst which was confirmed by endoscopy, and managed by endoscopic and percutaneous drainage. The patient was initially referred to our hospital for treatment of a pancreatic pseudocyst with hemorrhagic ascites and underwent endoscopic ultrasonographic‐guided stent placement. The next day, imaging demonstrated pneumoperitoneum and worsening ascites consistent with perforation, and the patient was treated conservatively. One week later, the patient developed severe abdominal pain. Endoscopy showed a large perforation site inside the pseudocyst connected to a large fluid collection and direct visualization inside the pseudocyst and fluid collection. The fluid collection was treated with percutaneous drainage, and the patient was discharged one week later with no complications.

## INTRODUCTION

Perforation of a pseudocyst is a rare lethal complication of pancreatic pseudocysts, occurring in less than 3% of all cases.^1^ While pancreatic pseudocysts can be managed electively, spontaneous perforation requires immediate treatment. Traditionally, surgical exploration has been the primary approach. However, recent reports have suggested that both endoscopic ultrasonography (EUS)‐guided and percutaneous drainages are viable treatment options for perforated pseudocysts.^2^ We present a rare case of spontaneous perforation of a pancreatic pseudocyst identified by endoscopy and successfully managed by endoscopic and percutaneous drainage.

## CASE PRESENTATION

A 54‐year‐old male with a history of chronic pancreatitis presented with upper abdominal pain to an outside hospital, where an abnormal peripancreatic mass was discovered on a computed tomography (CT) scan. He was subsequently referred to our hospital for further evaluation and treatment of the mass. The laboratory tests on admissions were as follows: white blood cell count 15,300/mm^3^, absolute neutrophil count 12,300/mm^3^, hemoglobin 10.4 g/dL, and platelets 522 × 10^9^/L.

**FIGURE 1:**
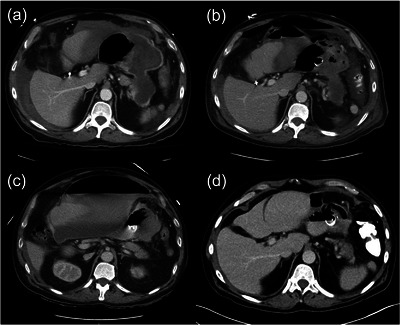
Serial computed tomography images (a) On admission, a peripancreatic pseudocyst with mild hemorrhagic ascites was observed. (b) On the third day, the pneumoperitoneum developed after lumen‐apposing metal stent cystgastrostomy. (c) On the ninth day, a new peritoneal fluid collection with free air became apparent. (d) Both the fluid collection and pseudocyst improved after percutaneous drainage on the seventeenth day.

A CT scan showed a 90‐mm large rim‐enhancing complex fluid collection arising from the pancreatic tail and extending along the greater curvature of the stomach, indicative of a pancreatic pseudocyst with thickened internal debris or hemorrhage. The CT scan also revealed complex hemorrhagic ascites in the peritoneum throughout the dependent portions of the abdomen and pelvis (Figure 1a), which was suspected to be pseudocyst fluid leakage, though no specific site of the perforation was confirmed at this time.

**FIGURE 2:**
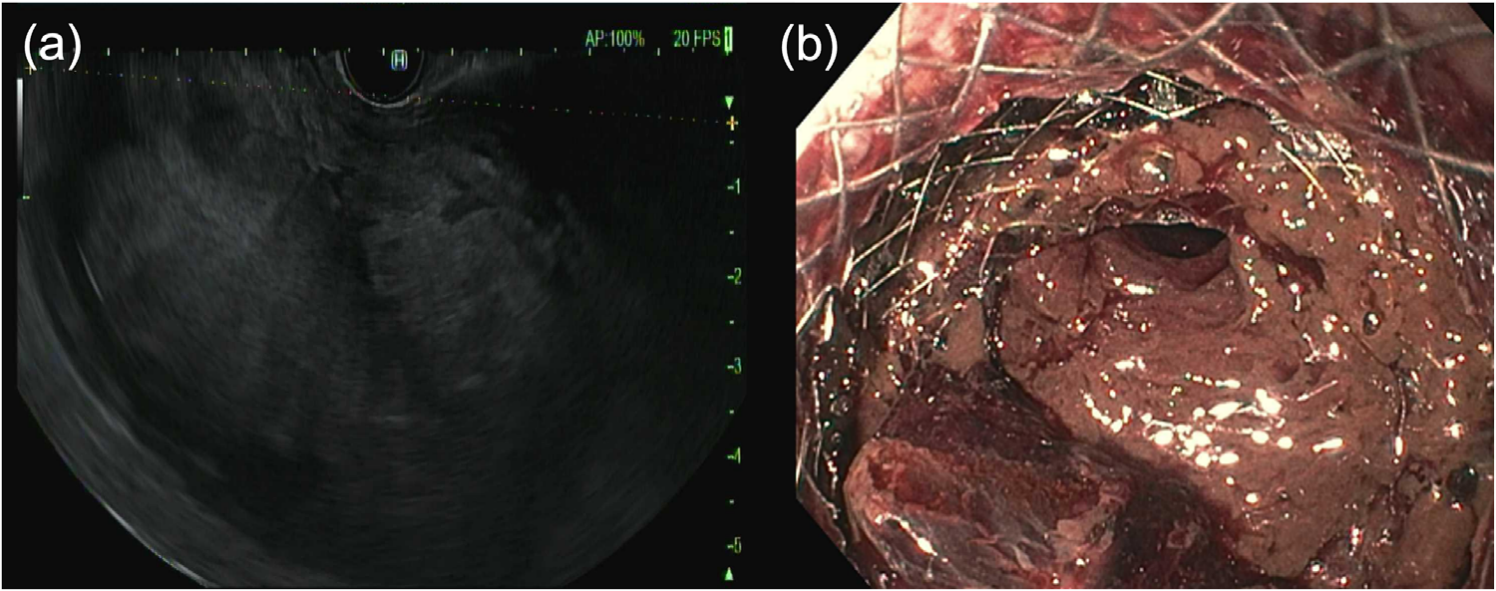
(a) Endoscopic ultrasonography showed evidence of a large pseudocyst with extensive hyperechoic material. (b) Lumen‐apposing metal stent was deployed by cystogastrostomy to reveal hemorrhagic necrotic material inside the pseudocyst.

On the second day of admission, EUS was performed, confirming the presence of a 90‐mm hypoechoic lesion with fluid and internal debris echogenicity at the tail of the pancreas consistent with a pancreatic pseudocyst. A 15 × 10‐mm lumen‐apposing metal stent (LAMS; HOT AXIOS system; Boston Scientific) was deployed for cystogastrostomy, subsequently dilated with 15‐mm balloon dilation, followed by hydrogen peroxide lavage, endoscopic irrigation, and removal of hemorrhagic materials and debris. (Figure 2). On the third day, a CT scan showed a slightly reduced size of the pancreatic pseudocyst but also revealed a minimal amount of pneumoperitoneum and increased hemorrhagic fluid collection around the liver (Figure 1b). The findings were suggestive of a spontaneous perforation of the pancreatic pseudocyst. At this time, our management plan was to proceed with conservative measures, with potential consideration for an additional endoscopic intervention approximately 1 week following the initial procedure.

**FIGURE 3:**
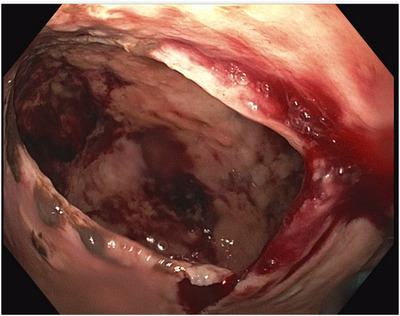
The gastroscope allowed direct vision of the perforation site in the pseudocyst connected to the new fluid collection.

On the ninth day, however, the patient developed an acute onset of severe abdominal pain, nausea, and neutrophilic leukocytosis (absolute neutrophil count 17,100/mm^3^). A CT scan showed a new giant 20 cm fluid collection containing hemorrhage and an extensive amount of free air along the greater omentum, communicating with the previously treated pseudocyst treated with the LAMS. The gastric lumen was sandwiched between the pseudocyst and the peritoneal fluid collection (Figure 1c). An emergent upper endoscopy was performed to verify that LAMS is properly positioned and to determine whether further endoscopic interventions are required for draining emerging peritoneal fluid collection (Video S1). When the gastroscope (GIF–H190; Olympus) entered the pseudocyst, a large perforation of the pseudocyst was identified and connected to a large fluid collection in the peritoneal cavity (Figure 3). No more necrotic tissue or debris was observed in the pseudocyst or the fluid collection. The fluid collection was drained percutaneously with a 14 Fr percutaneous drainage tube inserted from the anterior abdomen. On the seventeenth day, a CT scan showed a decreased fluid collection with no viable fluid and air (Figure 1d). The patient's diet was slowly advanced, and the patient was discharged on the twenty‐fifth day. On the forty‐sixth day, the LAMS and percutaneous drainage tube were successfully removed by endoscopy without complications in an outpatient setting.

## DISCUSSION

Spontaneous perforation of a hemorrhagic pancreatic pseudocyst is an exceedingly rare complication, but it can be extremely fatal if not managed promptly and properly. The perforation can either occur in the gastrointestinal tract lumen or the peritoneal cavity.^2^ Rupture into the gastrointestinal tract may lead to spontaneous regression of the pseudocyst as the pancreatic fluid is effectively drained and eliminated through the digestive system. However, rupture into the peritoneal cavity can result in severe complications such as peritonitis, the presence of free air, and hemorrhagic ascites with pancreatic fluid in the peritoneum.^3^ The exact mechanism underlying spontaneous rupture is not yet fully understood, although one hypothesis suggests that severe inflammation or the presence of activated lytic enzymes such as elastase and trypsin in the pseudocyst weakens the wall, leading to perforation.^4^ Despite the severity of this complication, there have been only a few reports documenting its diagnosis and treatment.

Early identification and diagnosis of perforation of pancreatic pseudocyst at an early stage can be challenging. Most case reports of perforated pancreatic pseudocysts rely on CT scans for diagnosis, which typically show a significant amount of free fluid and air in the peritoneal cavity. Additionally, physical examination findings indicative of peritonitis such as abdominal tenderness and guarding, along with signs of systemic inflammation and hemodynamic instability, can aid in the diagnostic process.^2^ In this particular case, mild hemorrhagic ascites were already observed in the initial CT scan before the EUS examination, suggesting a spontaneous perforation of the pseudocyst upon admission. Also, pneumoperitoneum was revealed on the next day after the LAMS placement. Spontaneous resolution of the pseudocyst was expected by LAMS placement, but unfortunately, the peritoneal large fluid collection was developed due to pseudocyst perforation. The upper endoscopy was able to directly visualize the actual site of the pseudocyst perforation, enter the newly developed peritoneal fluid collection, and confirm the absence of necrotic tissue.

In the literature, this is the first reported case of endoscopic visualization of an actual perforation extending to the perineum, providing valuable insights into the presentation and pathophysiology of this complication and its further management.

Traditionally, the management of a perforated pseudocyst involved emergent surgical exploration and external drainage, as leaving pancreatic fluid in the peritoneum could further exacerbate organ lysis.^5^ However, more recently, it has been recommended to consider EUS‐guided drainage and image‐guided percutaneous drainage as alternative treatment approaches. EUS‐guided drainage is often preferred as the primary treatment when the cystic fluid from the perforated pseudocyst is localized, reducing the likelihood of fluid backflow after pseudocyst drainage.^6^ Successful cases of EUS‐guided drainage of sterile fluid without the development of infections have been reported.^7^ Percutaneous drainage is primarily recommended for cases that cannot be effectively treated with endoscopy techniques or for those who fail to respond to endoscopic treatment, including those who are too critically ill to undergo endoscopic procedures.^8^ In such cases, self‐sealing of the pseudocyst is anticipated, and if it fails to occur after an extended period, surgical or endoscopic reevaluation becomes necessary. In this case, percutaneous drainage was performed, and an alternative option would have been to place another LAMS between the pseudocyst and the fluid collection. However, percutaneous drainage was deemed more suitable due to the high possibility of gastrointestinal fluid backflow into the peritoneal fluid collection through the LAMS and the perforation site, which would most likely cause prolonged drainage, while the percutaneous drainage tube provided effective external drainage of gastrointestinal fluid and air.^9^ There was also an option to add nasocystic drainage through the LAMS. However, this approach was deemed insufficient for effectively draining the newly formed large cavity, as nasocystic tubes could be easily clogged and could only provide slow drainage. The decision to proceed with percutaneous drainage was made by a careful discussion with our surgical team, interventional radiology team, and gastroenterology team. As predicted, the content in the new fluid collection was significantly decreased within one week using percutaneous drainage.

In conclusion, we report a rare case in which the site of perforation in a pancreatic pseudocyst was successfully visualized and examined by endoscopy. Percutaneous drainage effectively treated the new fluid collection caused by the perforated pseudocyst, while the pseudocyst itself was managed with the LAMS. This case highlights the usefulness of direct visualization of the pseudocyst perforation and the potential benefits of combined endoscopic and percutaneous drainage for managing perforated pancreatic pseudocysts.

## CONFLICT OF INTEREST STATEMENT

Dr. Yusuke Hashimoto is a consultant of FUJIFILM and has received honoraria from the American Society of Gastrointestinal Endoscopy.

## Supporting information

Video S1 Video of a CT scan and endoscopy on the ninth day, when the perforation site was confirmed.
